# A Quest of Great Importance-Developing a Broad Spectrum *Escherichia* *coli* Phage Collection

**DOI:** 10.3390/v11100899

**Published:** 2019-09-26

**Authors:** Joanna Kaczorowska, Eoghan Casey, Horst Neve, Charles M.A.P. Franz, Jean-Paul Noben, Gabriele A. Lugli, Marco Ventura, Douwe van Sinderen, Jennifer Mahony

**Affiliations:** 1 School of Microbiology and APC Microbiome Ireland, University College Cork, Western Road, T12 YT20 Cork, Ireland; j.m.kaczorowska@amc.uva.nl (J.K.); eoghan.casey@ucc.ie (E.C.); 2 Department of Microbiology and Biotechnology, Max Rubner-Institut, 24103 Kiel, Germany; horst.neve@mri.bund.de (H.N.);; 3 Biomedical Research Institute, Hasselt University, B-3590 Diepenbeek, Belgium; jeanpaul.noben@uhasselt.be; 4 Laboratory of Probiogenomics, Dept. Chemistry, Life Sciences and Environmental Sustainability, University of Parma, 43124 Parma, Italy; gabriele.lugli@genprobio.com (G.A.L.); marco.ventura@unipr.it (M.V.)

**Keywords:** bacteriophage, *Escherichia coli*, host range, phage therapy, *Shigella* ssp.

## Abstract

*Shigella* ssp. and enterotoxigenic *Escherichia* *coli* are the most common etiological agents of diarrheal diseases in malnourished children under five years of age in developing countries. The ever-growing issue of antibiotic resistance and the potential negative impact of antibiotic use on infant commensal microbiota are significant challenges to current therapeutic approaches. Bacteriophages (or phages) represent an alternative treatment that can be used to treat specific bacterial infections. In the present study, we screened water samples from both environmental and industrial sources for phages capable of infecting *E. coli* laboratory strains within our collection. Nineteen phages were isolatedand tested for their ability to infect strains within the ECOR collection and *E. coli* O157:H7 Δstx. Furthermore, since coliphages have been reported to cross-infect certain *Shigella* spp., we also evaluated the ability of the nineteen phages to infect a representative *Shigella sonnei* strain from our collection. Based on having distinct (although overlapping in some cases) host ranges, ten phage isolates were selected for genome sequence and morphological characterization. Together, these ten selected phages were shown to infect most of the ECOR library, with 61 of the 72 strains infected by at least one phage from our collection. Genome analysis of the ten phages allowed classification into five previously described genetic subgroups plus one previously underrepresented subgroup.

## 1. Introduction

10% of global child mortalities are caused by diarrheal diseases during the first five years of life [1,2]. Most of these deaths occur in so-called developing countries, particularly in sub-Saharan Africa and southern Asia [2,3]. The etiology of diarrheal diseases depends on a number of factors including the age of the child and the geographical location [3]. However, two groups of bacterial pathogens, *Shigella* spp. and enterotoxigenic *Escherichia coli* (ETEC) are predominantly associated with moderate-to-severe diarrhea in children irrespective of age or location [3]. There are only a small number of antimicrobials and particular measures, mainly pertaining to personal hygiene and food and water sanitization, that may prevent the spread of such bacterial infections and decrease the severity of the associated illness [4]. However, many *Shigella* and *E. coli* strains have become resistant to a variety of widely applied antimicrobials [5,6,7]. As it is more challenging to prevent infections in developing countries due to logistical problems pertaining to food and water sanitization, alternative treatments are urgently needed [8].

Bacteriophages (phages) are viruses which infect bacterial cells [9]. They can be purified to high titers and, as such, represent a possible alternative to conventional antibiotic therapies against bacterial infections [9,10,11]. One of the main advantages of phage therapy is the associated specificity of phages [10]. In contrast to antibiotics, which can cause collateral damage to the commensal microbiota of the patient, phages specifically target their host, infecting strains within closely related genera, thereby substantially limiting the negative impact on the commensal microbiota [10,12,13]. Conversely, the high specificity associated with phages may be equally problematic as it limits their potential therapeutic spectrum [12,13]. Phage cocktails, i.e., mixtures of different phages, have broader strain coverage than a single phage and are, therefore, often proposed for application in the treatment of complex bacterial infections involving many bacterial strains [12,13]. Phage cocktail composition can be edited in a flexible way in order to obtain a sufficiently broad strain coverage [13].

Phages are extremely abundant and widespread in nature, particularly in marine environments [14]. They are typically found in any environment in which their bacterial host is also present [15,16]. Phages infecting *E. coli* and its derivatives can be easily obtained from natural environments such as ponds, lakes, rivers, waste water, or human or animal fecal samples [17,18]. Within these environments one can identify a wide range of phages exhibiting distinct morphologies and host ranges, the latter allowing the formulation of various phage cocktails [17].

The attachment of a bacteriophage to a host receptor is an essential first step of an infection [19] and is mediated by the so-called “receptor binding protein” (RBP) located at the distal end of the phage tail [20]. In *Siphoviridae* phages, these proteins are components of tail spikes, large distal tail appendages termed baseplates, or fibers attached to a non-contractile tail, while in *Myoviridae* the RBPs are located within the components of the long and short tail fibers [21]. The RBP recognizes and binds to cell surface receptors [20,22,23], after which the phage attaches irreversibly to the cell surface and infection proceeds [23]. The interaction between bacterial receptor and phage RBPs is highly specific, and is therefore the primary determinant of the phage’s host range [21].

In the current study, we screened more than 50 samples from various environmental sources in order to isolate phages capable of infecting (prophage-free) laboratory *E. coli* strains. The isolated phages were subsequently tested against 72 strains of the ECOR collection, *E. coli* strain O157:H7 Δstx, and against *Shigella sonnei* strain 53G. The isolation of multiple phages was intended to allow the generation of a diverse phage collection with potential application as a broad host-range phage cocktail for the treatment of intestinal infections. The genomes of ten representative phages from this collection were sequenced to evaluate their potential for application in such phage cocktails. 

## 2. Materials and Methods

### 2.1. Bacterial Strains and Growth Conditions

Six strains of prophage-free *Escherichia coli* were used to isolate and propagate phages directly from environmental samples: BL21 [24], K12 [25], EC101, DH5α, XL1 Blue and Top10 (Thermo Fisher Scientific). ECOR, a collection of 72 *E. coli* strains [26], *E. coli* O157:H7 Δstx [27,28] and *Shigella sonnei* 53G [29] were then used to further assess the host range of isolated bacteriophages. *S. sonnei* 53G strain was grown in Brain Heart Infusion (BHI) broth (Oxoid, U.K.) at 37 °C without aeration. The ECOR collection of strains encompasses 72 reference strains of *E. coli* that is predicted to represent the phenotypic as well as genotypic diversity of the species [26]. It comprises strains of human, animal and plant origin and from a wide geographical spread (For details of the collection, see [26]. *E. coli* strains were grown in Luria-Bertani (LB) broth (1 %NaCl, 1 % tryptone and 0.5% yeast extract). *E. coli* strains were grown at 37 °C with aeration. All bacterial strains were preserved as glycerol stocks at −80 °C.

### 2.2. Phage Isolation from Environmental Samples

Water samples were obtained from both natural (over thirty samples collected from springs and rivers across counties of the Southern, Northern and Western provinces of Ireland, including for instance rivers and lakes in county Cork, Glencar Waterfall streams, Connemara National Park streams and puddles) and industrial (more than ten sewage water samples from manufacturing plants located in Belgium and in Ireland) sources. All water samples were filtered using 0.45 µm membrane filters in order to remove bacterial contamination. Chicken meat pieces were placed in a stomacher bag containing a minimal volume of SMG buffer (200 mM NaCl, 10 mM MgSO_4_ (Sigma Aldrich, Wicklow, Ireland), 50 mM Tris-HCl (Sigma Aldrich, Wicklow, Ireland), pH 7.5, 0.01 % gelatin (Sigma Aldrich) and homogenized using a stomacher. The samples were centrifuged at 5000× *g* for 10 min and the resulting supernatant was then filtered using 0.45 µm membrane. The presence or absence of bacteriophages was determined using the double agar layer method [30]. 100 µL of fresh prophage-free *E. coli* overnight culture was added to 4 mL of soft LB agar (0.6% agar) and overlaid on an LB agar plate. 150 µL and 300 µL of filtered water samples were pipetted on top of the bacterial layer. The plates were examined for the presence of plaques after overnight incubation at 37 °C. If necessary, samples were enriched in 1:1 ratio in double strength LB broth containing 1 % (*v/v*) overnight culture of the appropriate prophage-free strain. The samples were propagated overnight and plated using the double agar layer method as described above. Single plaques were propagated on an actively growing culture inoculated with 1 % culture and plaque-purified twice on the same host. Phage lysates were filtered using a 0.45 µm membrane and the titer was estimated using a previously described double layer titration method [30]. Phage dilutions were performed in sterile SMG buffer. Filtered lysates were stored at 4 °C until further use.

### 2.3. Plaque Morphology Analysis

A double agar layer method was used in order to obtain single plaques of the twice plaque purified phages. After overnight incubation at 37 °C, the plaques were measured and the pictures were taken using Gene Genius Bio-imaging System (Syngene, Cambridge, UK).

### 2.4. Host Range Determination

72 strains of the ECOR collection, as well as *E. coli* O157:H7 Δstx and *S. sonnei* 53G were used for host range analysis. 100 µL of the relevant overnight bacterial culture was added to 4 mL of molten LB soft agar and spread on LB plate. 10 µL of a given phage lysate with a minimum titer of 10^7^ pfu/mL was spotted on the surface of each plate. The plates were left to dry and incubated at 37 °C. The plates were examined after overnight incubation for lysis zones. All assays were performed in, at least, triplicate and in cases where the zones of lysis were hazy or did not produce individual plaques, the infectious capability was evaluated using plaque assays. Markov Cluster Algorithm clustering (MCL) of the host range was performed using MeV (Multi Experiment Viewer; http://mev.tm4.org/) software.

### 2.5. Phage Morphology Assessment

In order to perform an electron microscopic evaluation of the isolated phages, they were purified using a discontinuous caesium chloride density gradient ultra-centrifugation [31] and dialyzed against phage buffer (20 mM Tris-HCl (pH 7.2), 10 mM NaCl, 20 mM MgSO_4_) [32]. Adsorption of CsCl-purified phages to freshly prepared carbon film floated from a freshly coated mica sheet on a 400-mesh copper grid (Agar Scientific, Essex, UK) and negative staining with 2 % (*w/v*) uranyl acetate were performed as described previously [32]. Specimens were examined with a Tecnai 10 transmission electron microscope (FEI, Eindhoven, The Netherlands) operated at an acceleration voltage of 80 kV.

### 2.6. Phage DNA Isolation, Sequencing and Analysis of the Genomes

DNA isolation was performed using the Norgen Phage DNA isolation Kit (Norgen Biotek, ON, Canada) according to the manufacturer’s instructions. Purified DNA was sequenced using an Illumina MiSeq Sequencing System at the GenProbio facility (Parma, Italy). Genome assemblies of the paired end reads (2 × 250 bp reads) were performed with MIRA v4.0.2, while open reading frames (ORFs) were predicted with Prodigal v2.6. The ORFs were automatically annotated with BLAST (https://blast.ncbi.nlm.nih.gov/Blast.cgi) against NCBI and HMMER databases while functional analysis was performed by evaluation against the Pfam and HHPred databases [33,34]. The genomes were visualized and edited using Artemis Release 15.0.0 (http://www.sanger.ac.uk/science/tools/artemis) and nucleotide BLAST (BLASTn). The percentage of similarity between the phage proteins was acquired using protein BLAST (BLASTp).

### 2.7. Proteomic Analysis of JK16

Phage particles of JK16 phage were purified (see above) and phage proteins were concentrated using methanol-chloroform precipitation [32]. Phage proteins were separated using SDS-PAGE on a 12.5 % polyacrylamide gel. The gel was stained with 0.25 % Coomassie blue. Protein bands were excised from the gel, de-stained and the proteins were digested using trypsin-Gold [32]. The samples were analyzed using electrospray ionization-tandem mass spectrometry (ESI-MS/MS), as described previously [32].

## 3. Results

### 3.1. Phage Isolation, Plaque Morphology and Host Range Determination

Forty one phages were isolated from water samples. Additionally, five phages were isolated from one sample of chicken meat using the prophage-free laboratory strains in the primary screen. The host range of these isolates was assessed employing the ECOR72 strain collection. A substantial number (twenty five) of phages displayed identical host range profiles to at least one of the other phages of the collection, and they were therefore not further investigated except for one representative phage of each of the 19 host range groups was selected for further characterization (Table 1). The phages were also shown to exhibit a variety of plaque morphologies with the majority displaying medium sized plaques with a diameter of ≤ 3 mm. Four phages (JK27, JK28, CM1 and CM8) formed tiny, regular plaques of 0.5 mm diameter, while JK16 and JK42 formed large plaques (up to 4 mm diameter) with an obvious “halo” surrounding the plaque in the case of JK16. The plaque morphologies of individual phage isolates on susceptible strains were not observed to differ significantly in size or appearance and the typical morphologies are presented in Table 1 on the relevant isolation host strain.

**Figure 1:**
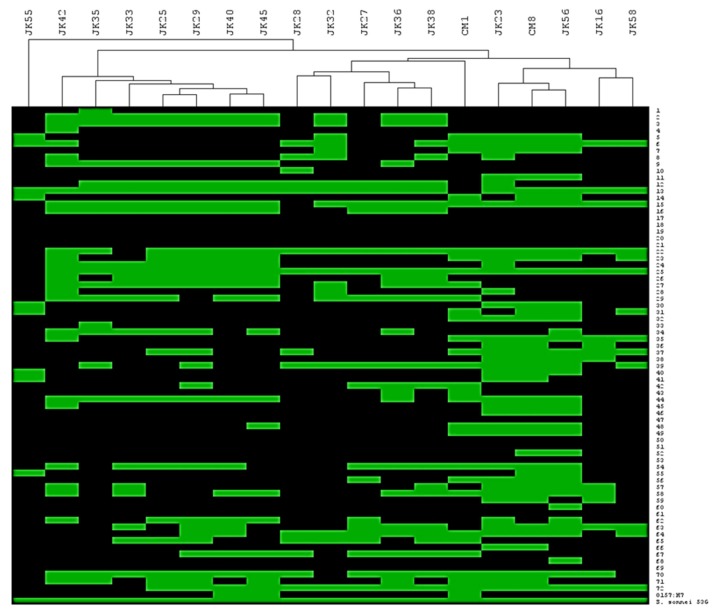
Hierarchical clustering of isolated phages depending on their host range; 72 ECOR strains, *E. coli* O157:H7 Δstx strain and *S. sonnei* 53G strain; green box represents successful phage infection and black box - no infection.

The host range of the phage collection was ascertained on a panel of 73 *E. coli* strains as well as a single *Shigella sonnei* strain. Table 1 highlights that the phage isolates lyse 15-54 % of the panel of strains in our collection. To assess if the host range profiles were unique/overlapping and if patterns of infection could be discerned, a heat-map of the infection profiles was generated. Based on the identification of distinct/overlapping host range profiles, the collection of nineteen phages was divided into three main groups (Figure 1). The first cluster was represented by JK28, JK32, JK27, JK36 and JK38. Three phages of this cluster (i.e., JK27, JK28 and JK32) had been isolated from a waterfall stream, while two phages (JK36 and JK38) originated from sewage water. The second cluster is represented by seven phages which had been isolated from sewage water or a waterfall stream: JK42, JK35, JK33, JK25, JK29, JK40 and JK45. Two phages isolated from chicken meat (i.e., CM1 and CM8) make up the third cluster, together with environmental water isolates (JK23 and JK16), and sewage isolates (JK56 and JK58). Based on the heat-map presented in Figure 1, CM1 appears to be intermediate between the cluster 1 and 3 phages mentioned above. However, CM1 shares 15 host strains with cluster 3 phages and only eight shared hosts with those of cluster 1 phages. Therefore, based on host range it is grouped among the cluster 3 phages. Another sewage isolate, phage JK55, had a very different host range from all other phages and is thus considered distinct based on its host range (Figure 1). Phage infectivity (i.e., number of infected strains divided by total number of tested strains x 100 %) ranged from 14 % (JK55) to 54 % (JK23) (Table 1). Altogether, the tested phages infected all but 11 ECOR strains (Figure 1). All tested phages infected *S. sonnei* 53G (Figure 1).

### 3.2. Phage Particle Morphology

**Figure 2:**
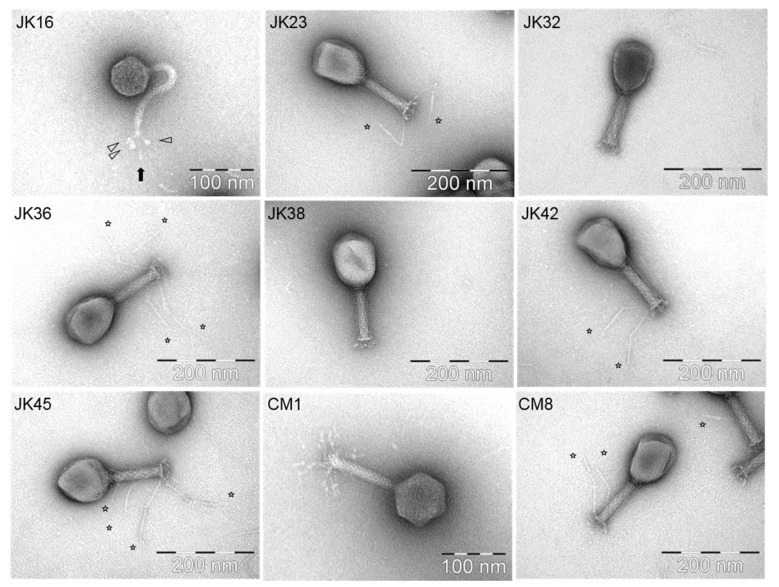
TEM morphologies of selected phages. Each phage name is indicated in the upper left corner of the picture while the scale is mentioned in the lower right. The star signs indicate the position of long tail fibers of the phages, while the arrow shapes in the JK16 picture point out the globular structures.

Ten out of the nineteen selected phages (at least one phage representing each of the identified host range groups) were selected for particle morphology assessment by electron microscopy. The phages were observed to belong to one of two morphological groups [35] (Table 2). With the exception of JK16, all isolates belonged to the *Myoviridae* family, possessing long contractile tails [35]. JK23, JK32, JK36, JK38, JK42, JK45 and CM8 exhibited a T4-like morphology (Figure 2) and displayed similar particle dimensions (Table 2). Phage JK36 possesses exceptionally long baseplate fibers, exceeding 160 nm in length (Figure 2, Table 2). Conversely, phage CM1 possesses smaller capsid and baseplate fibers compared to the other *Myoviridae* phage isolates (Figure 2, Table 2). JK55 particles were damaged despite several attempts therefore, morphological data pertaining to this phage is not available (Table 2).

The only *Siphoviridae* phage isolate observed in this study, JK16, had a very flexible, non-contractile tail with (at least) three long tail fibers with distal globular structures (Figure 2). The capsid size varied from 63.7 to 64.7 nm, while the tail length exceeded 150 nm (Table 2). The unique distal globular structures varied slightly in size depending on their localization–the size of the globular structure located beneath the distal tail end ranged from 9.0 ± 1.3 nm × 8.1 ± 0.9 nm (indicated with a black arrow in Figure 2), while similar structures present on short side fibers measured 11.1 ± 1.5 nm × 7.0 ± 1.1 nm (indicated with white arrows in Figure 2).

### 3.3. Identification of Phage Genetic Lineages

In order to investigate the genetic diversity of the isolated phages, the genomes of the ten selected isolates were sequenced and analyzed. All isolates possessed double-stranded DNA genomes with the majority displaying identity to previously described phages with six distinct genetic subgroups of phages identified based on the similarity to the closest database relatives acquired by BLASTn analysis of the whole genome sequences. Three subgroups of phage genomes related to the T-even group: (i) T4-even, (ii) RB69-even and (iii) pseudo-T-even. The fourth group, (iv) with an rV5-like genome, was represented by phage CM1. The phage with the narrowest host range, i.e., JK55, proved to be a close relative of *Salmonella* phage Felix O1 and was categorized in subgroup (v) – the Felix O1-like subgroup. JK16 (assigned to new subgroup vi) showed no resemblance to any broadly described phage group, and thus it was further investigated in more detail as will be described later.

Comparative whole genome analysis was performed between the subgroups but also within the subgroups. However a focused analysis of the receptor binding proteins (RBP) encoded by the phage isolates was also undertaken using BlastP analysis to reflect the diversity of host interactions as it is the among the most diverse genomic regions within the phage subgroups. In the case of T-even phages, the tail structural region and the receptor binding location have been described previously in detail [23], thus we identified the region based on the similarity to already known tail structural regions. For the rest of the phage groups, HHpred analysis was employed to assign potential RBP functions based on structural homology predictions [33].

### 3.4. T-Even Phages: T4, RB69 and Pseudo-T-Even (RB49-like) Subgroups

**Figure 3:**
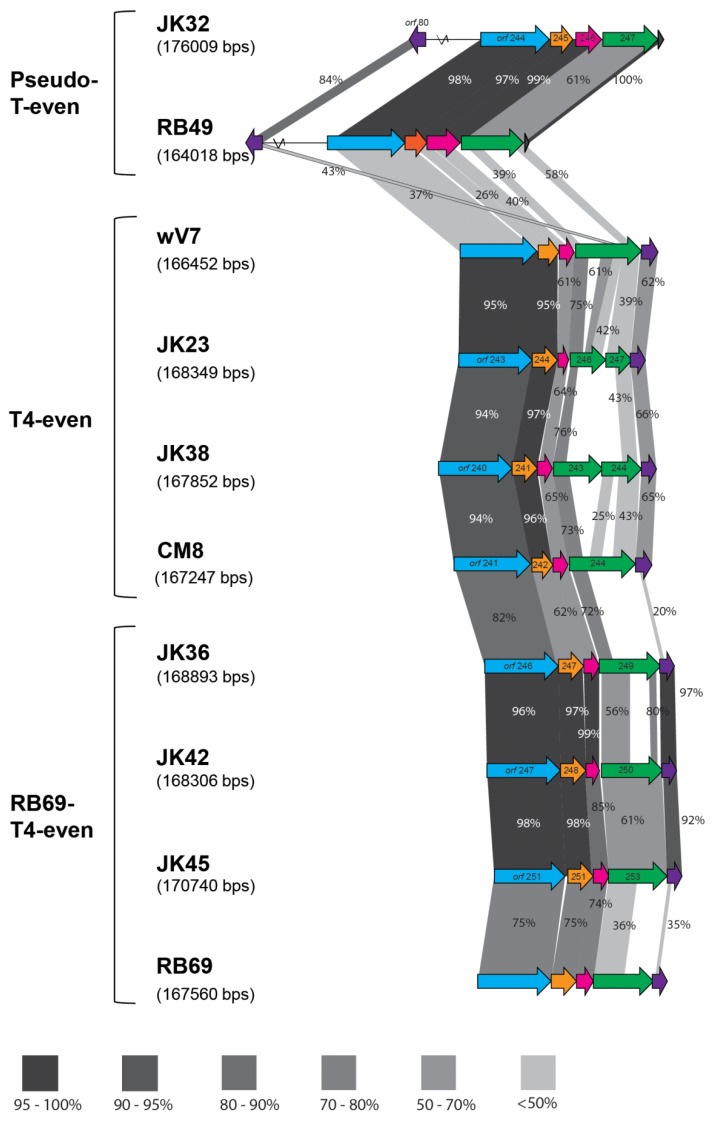
Comparison of the tail fiber genes of the T-even phages; the percent values of the similarity between the structural proteins were acquired by BLASTp; blue arrow represents the gp34 proximal fiber, orange–gp35 tail fiber hinge, pink–gp36 small distal tail fiber subunit, green–gp37 large distal tail fiber subunit and purple–gp38 tail fiber adhesin. The dark blue arrow present in the pseudo-T-evens indicates the Dc5 ORF. Different tones of grayscale represent different range of protein similarity between the phages. Absence of shaded regions is indicative of ORFs with no shared similarity. All-against-all BlastP analysis was first undertaken to identify the isolates with the most similarity and they are placed in order of similarity in the figure above. In this comparison, both phages isolated in this paper and reference phages (RB49, wV7 and RB69) are compared. Percentage similarity values presented indicate the similarity of the proteins between the two neighboring isolates.

Seven of the sequenced phages showed similarity to T-even phages (Table 1 and Table 3). The diversity of the T-even family is remarkable: comparisons between various isolates with distinct host ranges show a high degree of diversity in the hypervariable regions [36,37]. The patchwork-like genomes of these viruses consist of stretches of high variable regions around a conserved core [38]. As mentioned above, we differentiated three subgroups of our T-even phages - T4-even, RB69-T4-even and pseudo-T-even. Three out of ten sequenced phages (JK23, JK38 and CM8) showed over 80 % nucleotide similarity to a strain of a T4 phage (Genbank accession no. KJ477684.1) [39], and over 90 % nucleotide similarity to *E.coli* phage wV7 (Genbank accession no. HM997020.1). Therefore, these phages were classified among the T4-even subgroup. All phages possessed similar genome sizes of almost 170 kb, with a low G+C content (Table 3), which is typical for T4 phages [40,41]. Three phages - JK36, JK42 and JK45 - displayed high nucleotide similarity to phage RB69 (Genbank accession no. NC_004928.1). T4 and RB69 share approximately 80 % orthologous genes showing more than 80 % similarity [41]. These phages had similar G+C content, and a similar genome size (Table 3). JK32 possessed the largest genome, exceeding 176 kb, with an average G+C content of 40 % (Table 3). BLASTn against the NCBI database showed similarity of this phage to phage RB49 (Genbank accession no. NC_005066.1), which has been classified as a “pseudo-T-even” phage [37]. Figure 3 illustrates the differences in the RBP region organization of the T-even phages.

### 3.5. rV5-Like Subgroup

The chicken meat isolate CM1 displayed no similarity to any of the T4 derivatives, while BLASTn analysis revealed similarity to the *Enterobacteria* phage vB_EcoM-FV3 (Genbank accession no. JQ031132.1) and *E. coli* bacteriophage rV5 (Genbank accession no. DQ832317.1). Based on this analysis, the phage was classified in “rV5-like” subgroup, which was recently defined [42]. CM1 has a genome size of nearly 140 kb, with a higher G+C content than all T4-related phages (Table 3). Phage rV5 is a derivative of phage V5, one of the phages employed in *E. coli* O157:H7 typing [43]. The genomics of rV5-like phages has been described previously [42,43]. HHpred analysis was employed to identify the likely RBP of CM1 [33] (Supplementary Table S1). The protein product of the downstream located gene, *orf*40 (encoding a protein of 1266 aa), displays significant similarity to the L-shaped tail fibre protein of phage T5 based on HHpred analysis (Probability = 99.94, E-value = 1.7e-10) (Table S1). This tail fiber is predicted to contain receptor binding domains and is responsible for early host recognition, akin to GP37 in T4 phage [44]. The C-terminus of this *orf*40-encoded protein, exhibits similarity to a chaperone of *Enterobacteria* phage K1F (Probability = 99.13, E-value = 1e-12) (Table S1), which is responsible for the correct folding of tail proteins [45]. Another domain located in the C-terminus showed high similarity (Probability = 99.25, E-value = 7e-14) to RBP of *Salmonella* phage vB_SenMS16. Therefore, we assume that receptor binding region is likely encoded by *orf*40.

### 3.6. Felix O1-Like Subgroup

JK55 exhibits a relatively small genome (86,219 bp) compared to the isolates mentioned above, with 124 predicted ORFs (Table 3). This phage displays significant similarity to Felix O1 (98 % similarity, 93 % coverage), a phage mainly known for its use in typing *Salmonella* [46]. The genome characteristics of Felix O1 have been described previously [46]. HHpred analysis revealed that the product of *orf*74 of JK55 phage exhibits similarity to T-even’s gp37 (Probability = 99.43, E-value = 1.5e-15), a T-even RBP that was described in detail in previous sections. The *orf*74 protein product is therefore proposed to represent the RBP of phage JK55.

### 3.7. Siphophage JK16 Genome and Structural Proteome

**Figure 4:**
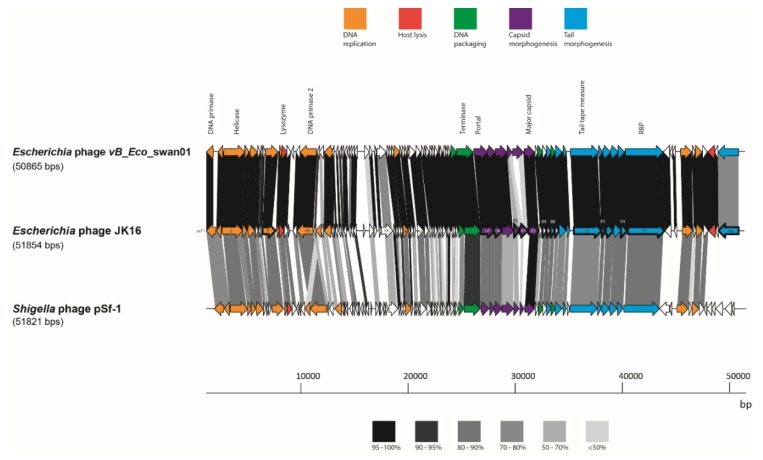
Genome maps of JK16 phage and its close relatives, vB_Eco_swan01 and pSf-1. The putative functional regions are indicated with the colors described above. ORF sharing amino acid identity are linked by grey shading. The degree of sequence sharing is described by the shading scale in the bottom of the figure. The ORFs of JK16 phage that have been identified as structural components using ESI-MS/MS are outlined with a thick line.

The JK16 genome is substantially smaller than any of the other nine sequenced genomes from the current phage collection, measuring almost 52 kb and encompassing 84 predicted ORFs (Table 3 and Table S2). JK16 exhibits the highest G + C content of all analyzed phages, at approximately 45 % (Table 3). This phage genome exhibits high sequence similarity to just three phages - *Escherichia* phage vB_Eco_swan01 (98.36 % similarity, 88 % coverage; Genbank accession no. LT841304.1); *Escherichia* phage SECphi27 (98.04 % similarity, 88 % coverage; Genbank accession no. LT961732.1); and a *Shigella* phage pSf-1 (77.6 % similarity, 73 % coverage; Genbank accession no. KC710998.1) [47]. The sequence of JK16 was annotated and analyzed using HMMER and BLASTp (Supplementary Table S2) resulting in the assignment of the potential functions of 36 ORFs using this method. Four functional clusters can be identified on the genome of JK16: (1) DNA metabolism and replication, (2) DNA-packaging, (3) cell lysis and (4) morphogenesis genes (head and tail genes) (Figure 4).

The structural region encoding the capsid elements encompasses at least five genes (*orf*58-62). Based on HHpred, Pfam and BlastP analysis, we suggest that *orfs* 58-61 encode head stabilization, scaffolding and/or decoration proteins while orf62 encodes the major capsid protein. This protein bears structural homology with the major capsid protein of the *Pseudoalteromonas* phage TW1 (100 % probability, PDB5WK1_E). SDS-PAGE analysis of the structural proteome of JK16 identified three proteins of high abundance. One of these, which runs at approximately 28 kDa may represent the mature and cleaved version of the major capsid protein which is predicted to be 35.9 kDa in its’ unprocessed form (Table 4 and Figure S1). There are three ORFs between the head and tail structural component-encoding regions (*orf63-*65) for which functions could not be readily assigned. However, HHpred and Pfam analysis suggests that *orfs 64/*65 bear some structural/sequence homology to head-tail connector proteins of the coliphage HK97 (77.2 % PF05135.13) and *Shigella flexneri* (95.3 % PDB entry 2K24_A). Ten genes can readily be associated with tail morphogenesis (*orf*66, *orf*68-75 and *orf*84). Detailed analysis using HHpred revealed predicted specific functions of several of the structural gene products (Supplementary Table S3). For example, *orfs67* and 69 are predicted to encode tail assembly chaperone proteins while *orf68* likely acts as the major tail protein. Based on SDS-PAGE analysis of the structural proteome of JK16, there is a protein of high relative abundance of approximately 25 kDa, which is in keeping with the predicted mass (24.4 kDa) of Orf68 (Fig. S1). It is proposed that orf73 may function as the tail associated lysin with identifiable hydrolytic domains observed in this protein. Finally, the product encoded by *orf*75, which represents the largest protein of JK16 (1192 aa), exhibits similarity to various adhesion domains (data not shown), and also to a tail domain of bacteriophage MuSo2 (Table S3), and based on these similarities it is the most likely candidate to represent the RBP of this phage. Interestingly, *orf*84, located a few genes downstream from the tail structural region, encodes a putative tail protein (Table S2). Interestingly, the SDS-PAGE profile of JK16 indicates the presence of a third highly abundant protein, which cannot be readily attributed a function based on our analysis. However, based on its size, we suggest that it is likely a capsid stabilization protein.

The ESI-MS/MS was performed in order to recognize and characterize the JK16 proteins present/identified in purified virions. This analysis identified 16 JK16 structural proteins, which are presented in Table 4. Most of the predicted proteins were located within the structural region (tail and head proteins) or were associated with the DNA packaging module (portal proteins) (Table 4). Almost all tail structural region proteins were detected by the ESI-MS/MS analysis, with the exception of the putative major tail protein (encoded by *orf*68), tail assembly chaperone (*orf*69) and tail assembly protein (*orf*73), the latter two being likely non-structural components while it is unclear why the major tail protein was undetected in this study. This analysis identified 15 JK16 structural proteins, which are presented in Table 4. Most of the predicted proteins were head and tail proteins whose genes were located within the virion morphogenesis region of the genome, which is immediately downstream of the DNA packaging genes (Figure 4, Table 4). An additional protein, gp9 was identified by mass spectrometry, which is unlikely to be a virion structural protein due to its homology to phosphodiesterases. We infer that this protein co-purified with JK16 virions and was identified due to the sensitivity of the mass spectrometry analyses, however further analyses are required to determine the role of gp9 in the viral life cycle. It is also notable that the detection of this protein was at levels just above the threshold values for the number of detected peptides and the coverage level.

The complete proteome of JK16 was compared with protein sequences of its close relatives, *Escherichia* phage vB_Eco_swan01 and *Shigella* phage pSf-1 (Figure 4). There were only minor regions of significant divergence from *Escherichia* phage vB_Eco_swan01 including hypothetical proteins (orf18, orf30 and orf33), while elements within the structural module were also observed to exhibit divergence: orf60 (a putative HtjA), orf61 (scaffolding protein) and orf84 (tail structural protein).

## 4. Discussion

Bacteriophages are progressively being acknowledged as a potential tool to control pathogens, including *Shigella* ssp., both in food and in human infections [48,49,50,51,52]. It is a distinct advantage that phages can be relatively easily isolated from a range of environments. In this study we focused on bacteriophage isolation from diverse environments: industrial (sewage), commercial/animal (poultry) and natural (natural environmental water reservoirs). In each sample type a variety of phages were isolated, representing different (sub) groups. The most prevalent group of phages was the T4-even group, i.e., members of this group were present in all sample types.

Screening for individual phages is a demanding quest, as there is no quick selection for isolates which are different from each other. Here, we used a three-step procedure, which increases the chance of selecting distinctly different phage isolates. Firstly, we selected phages based on varying plaque morphologies, and secondly differentiation was made based on varying host ranges. Finally, ten of the phages were categorized into subgroups using genome sequence analysis. For selection for potential therapeutic application, the phages used in the cocktail should ideally be strictly lytic [9,52]. As all the phages were double plaque purified and propagated on prophage-free laboratory strains, the lysates can be considered pure and free from prophage contamination from the propagating strains. Phage isolation on prophage-free, well-described laboratory strains, and the advantages of such procedures have been described previously [53,54]. Therefore, we pursued this strategy in this study to ensure the most suitable approach to identify and propagate pure and single phage preparations to ensure that induced prophages would not contribute to the observed host ranges.

Host range is a major property for successful phage treatment, as it defines the therapeutic spectrum [10,55]. Phages within our collection showed various host range patterns and various infectivity, ranging from 14 % to 54 % of the tested strains (Figure 1). The phage having the narrowest host range (JK55) proved to be a member of Felix O1 subgroup, which infects *Salmonella* strains. Thus, the phage collection might have potential use for a variety of applications, both in specific, single-origin infections and also complex infections caused by many different bacterial strains. The finding that our phage collection was able to infect our reference *Shigella sonnei* strain, as well as the majority of the ECOR library (Figure 1), suggests that these phages will be effective against various serotypes of *Shigella* ssp., ETEC strains, *Salmonella* strains (especially JK55 and CM1 phages) and potentially also more distant members of enterobacteria, such as *Klebsiella* [56].

Ten phages from the obtained collection were subjected to morphological and genomic analysis. Almost all identified phages belonged to the *Myoviridae* family, with JK16 being the only phage that was shown to belong to the *Siphoviridae* family. Genomic analysis revealed that seven out of ten phages showed close resemblance to T-even phages. These T-even-like phages were shown to display the highest divergence in their RBP region, which determines their host range, indeed being consistent with their experimentally determined unique host range profiles (Figure 1 and Figure 3). The only *Siphoviridae* phage, JK16, was genetically and morphologically distinct to the rest of the isolated phages. One of the most closely related phages to JK16, vB_Eco_swan01, was previously suggested to represent a new bacteriophage species among the *Tunavirinae* [57] while SECphi27 was also identified as a likely Siphovirus based on identity to vB_Eco_swan01 [58]. However, these phages were not characterized beyond the level of their genomes. Here, we examined the morphology of the phage and confirmed its structural protein content by mass spectrometry defining this phage as a member of a novel group of that may present a useful addition to phage cocktails given their distinct morphology, genome characteristics and likely host interactions. No lysogeny-related functions, such as integrases or repressors, were observed in the genomes of the 19 sequenced isolates, which is of importance if such phage isolates are to be employed in phage-based therapies [15]. Importantly, we did not detect any virulence factors or antibiotic resistance genes. These genes are highly undesirable if phages are to be used for therapeutic purposes, as they can be transferred to the bacterial host and cause adverse effects of the therapy [15].

Phage diversity within a cocktail is highly desired, as distinct phages may interact with various bacterial receptors, thereby expanding the therapeutic spectrum. The wide variety of phage-recognized receptors of Gram-negative bacteria has previously been described [19,59]. Outer-OmpC is known to be an important protein receptor of T4-like phages, since absence of this receptor in the host results in decreased infectivity [59]. The Gp37 tail fiber protein of T4 phage possesses a histidine-rich, unique region, which is involved in host recognition [59]. T4 phages also recognize rough type (R-type) lipopolysaccharide (LPS) receptors, which are common in *Shigella* ssp. [59]. The R-type LPS receptors lack the so-called O-antigen, which is a high-variable region [21,59]. Therefore, the host range of R-type LPS recognizing phages is broader than those recognizing the smooth-type (S-type) LPS, which possesses this hypervariable region [21,59]. Felix O1 phages are LPS-specific [60]. As phage JK55, analyzed in this paper, showed a very narrow host range, we speculate that this particular phage has preference for the S-type LPS. It remains uncertain, how the novel JK16 phage binds to the host cell and which bacterial receptors it recognizes. Given its distinctive morphology and unusual genome organization, it is possible that this phage recognizes a different bacterial receptor. For instance, some of the phages are able to bind to distal part of bacterial flagella [59], and it seems to be quite a common binding site for enterobacteria-infecting *Siphoviridae* [19].

Owing to the genetic diversity, the strictly lytic nature of the isolates and broad spectrum of infection, we feel that this phage collection has therapeutic potential. It is aimed to develop and optimize a broad-range phage cocktail which can corroborate the concept of phage therapy against *Shigella* ssp. and ETEC infections. Further work is currently being undertaken to assess the antimicrobial properties of such a phage cocktail.

## Figures and Tables

**Table 1:** Host range and plaque morphology of the 19 phages examined in this paper. Phages were isolated from various environments: natural springs, industrial waste water and chicken meat. The infectivity of the phages was calculated based on the host range (Figure 1). Phages exhibited various plaque morphologies, varying from very small (less than 0.5 mm diameter) to large (up to 4 mm diameter). Plaques were formed on agar plates with *E. coli* host that was used to isolate the phages.

Phage	Source of Isolation	Isolation *E. coli* Host	Infectivity (% of Infected Strains)	Plaque Morphology and Diameter (mm)
JK16	Cork City stream	DH5α	22	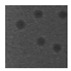 3–4
JK23	Connemara National Park stream	K12	54	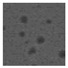 2–3
JK25	Glencar Waterfall	DH5α	32	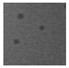 2–3
JK27	Glencar Waterfall	BL21	26	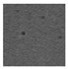 0.5–1
JK28	Glencar Waterfall	BL21	20	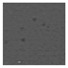 0.5–1
JK29	Glencar Waterfall	Top10	38	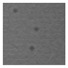 1–2
JK32	Glencar Waterfall	XL1 Blue	26	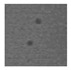 1–2
JK33	Glencar Waterfall	K12	28	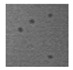 0.5–1
JK35	Sewage (Ireland)	DH5α	27	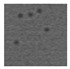 1–2
JK36	Sewage (Ireland)	Top10	35	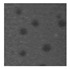 2–3
JK38	Sewage (Ireland)	BL21	34	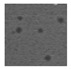 1–2
JK40	Sewage (Ireland)	XL1 Blue	34	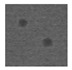 2–3
JK42	Sewage (Ireland)	DH5α	38	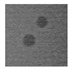 3–4
JK45	Sewage (Ireland)	DH5α	34	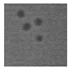 2–3
JK55	Sewage (Aaalst, Belgium)	DH5α	14	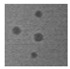 2–3
JK56	Sewage (Aaalst, Belgium)	DH5α	51	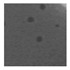 1–2
JK58	Sewage (Aaalst, Belgium)	K12	19	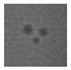 2–3
CM1	Chicken meat	DH5α	42	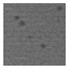 0.5–1
CM8	Chicken meat	BL21	53	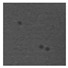 0.5–1

**Table 2:** Morphologies of the selected phages and measurements of the particles; Abbreviations: tl - tail length (incl. baseplate), tw - tail width, hl – head length, hw – head width, bpw – baseplate width, bps – baseplate spikes length, fbf – free baseplate fibers length; the number of measured phages is stated in brackets. N.A. – the measurement is not available.

Phage	Classification	Tail (nm)	Head (nm)	Baseplate (nm)
JK16	*Siphoviridae*	tl 151 ± 7 (8) tw 13± 1 (8)	hw 64 ± 1 (8)	-
JK23	*Myoviridae*	tl 106 ± 3 (10) tw 22 ± 1 (10)	hl 115 ± 3 (10) hw 89 ± 5 (10)	bpw 31 ± 2 (9) bps 15 ± 1 (8) fbf 125 ± 1 (3)
JK32	*Myoviridae*	tl 107 ± 3 (7) tw 24 ± 2 (7)	hl 114 ± 3 (7) hw 83 ± 2 (7)	bpw 32 ± 2 (6) bps 13 ± 1 (6) fbf N.A.
JK36	*Myoviridae*	tl 111 ± 1 (7) tw 25 ± 1 (7)	hl 112 ± 4 (7) hw 82 ± 2 (7)	bpw 32 ± 3 (9) bps 13 ± 2 (7) fbf 167 ± 28 (5)
JK38	*Myoviridae*	tl 107 ± 4 (8) tw 23 ± 1 (6)	hl 115 ± 3 (8) hw 86 ± 5 (8)	bpw 27 ± 2 (8) bps 13 ± 2 (7) fbf N.A.
JK42	*Myoviridae*	tl 107 ± 3 (10) tw 22 ± 1 (10)	hl 112 ± 3 (10) hw 82 ± 5 (10)	bpw 33 ± 3 (11) bps 14 ± 2 (7) fbf 146 ± 13 (14)
JK45	*Myoviridae*	tl 109 ± 2 (13) tw 23 ± 1 (13)	hl 117 ± 3 (13) hw 88 ± 2 (13)	bpw 32 ± 2 (11) bps 17 ± 1 (7) fbf 144 ± 18 (18)
JK55	*Myoviridae*	Damaged particles; N.A.		
CM1	*Myoviridae*	tl 107 ± 6 (7) tw 20 ± 2 (7)	hw 85 ± 2 (7)	bpw 28 ± 5 (6) bps N.A. fbf 43 ± 5 (13)
CM8	*Myoviridae*	tl 108 ± 1(9) tw 22 ± 1 (9)	hl 114 ± 4 (9) hw 84 ± 2 (9)	bpw 33 ± 2 (9) bps 17 ± 1 (9) fbf 126 ± 11 (8)

**Table 3:** Genome characteristics and Genbank accession information of the ten sequenced isolates in this study.

Phage	Accession Number (GenBank)	Group	Genome Size [bp]	Number of Predicted ORFs	Average GC Content [%]
JK16	MK962751	New siphovirus group	51,854	84	44.55
JK23	MK962752	T4-even	168,349	272	35.32
JK32	MK962753	Pseudo-T-even	176,009	269	40.40
JK36	MK962754	RB69-like	168,893	270	37.73
JK38	MK962755	T4-even	167,852	268	35.48
JK42	MK962756	RB69-like	168,306	271	37.58
JK45	MK962757	RB69-like	170,740	273	37.64
JK55	MK962758	FelixO1-like	86,219	124	38.96
CM1	MK962749	rV5-like	139,598	217	43.54
CM8	MK962750	T4-even	167,247	269	35.28

**Table 4:** JK16 Phage proteins as identified by ESI-MS/MS; putative functions were estimated using HMMER, BLASTp or HHpred. The threshold was at least 2 single independent peptides or 5% coverage value. The no. of peptides identifying the proteins and the % coverage of the proteins as well as the predicted molecular mass of the proteins and the no. of their component amino acids identified are presented below.

ORF	Putative Function	No. of Peptides	No. Identified Amino Acids	Coverage (%)	Predicted Molecular Mass (kda)
9	Phosphodiesterase	3	31	7.4	47.8
57	Portal	13	188	43.7	48.5
58	Head morphogenesis	2	21	8.4	28.7
60	HtjA; preneck appendage	14	144	85.2	17.9
61	Scaffolding protein	8	74	28.5	28.2
62	Major capsid	16	192	59.4	35.9
64	Hypothetical protein	4	41	29.5	15.9
65	Head-tail connector	4	44	35.5	13.7
66	Tail protein	3	27	18.4	16.4
67	Hypothetical protein	7	92	41.8	15.2
70	Tail measure protein	25	305	34.2	98
71	Tail protein	5	57	49.1	12.9
72	Tail tip assembly / minor tail protein	3	41	16.2	28.4
74	Tail assembly protein	1	17	8.6	20.8
75	Tail protein / RBP	18	191	16.02	132.1
84	Tail protein	20	187	28.7	70.7

## Supplementary Materials

The following are available online at https://www.mdpi.com/1999-4915/11/10/899/s1, Figure S1: SDS-PAGE analysis of JK16 proteome, Table S1: Putative functions of CM1 tail structural proteins based on HHpred analysis, Table S2: Genes and predicted gene products (predicted by HMMER and BLASTp analysis) of the novel phage JK16 and S3, Table S3: Putative functions of a few of the JK16 tail structural proteins.
