# Supplementary material for: A Quest of Great Importance-Developing a Broad Spectrum Escherichia coli Phage Collection
Source: Viruses. 2019 Sep 26;11(10):899. doi: 10.3390/v11100899 (PMC6832132; doi:10.3390/v11100899)
Supplement: Supplementary file 1 [file viruses-11-00899-s001.zip › 7-viruses-533017-suppl/Supplementary files R1.pdf]

Supplementary files

Table S1. Putative functions of CM1 tail structural proteins based on HHpred analysis.

orf no.	Product size (number of aa)	Putative function	Organism / virus	ID	Aligned positions	Probability	E-value
36	1199	Tail protein (proximal tail?)	*Enterobacteria* phage Mu	1WRU_A	192	95.31	0.19
37	141	Lysin	*Streptococcus pyogenes*	5UDN_B	87	85.04	11
38	183	Tail protein (hinge?)	*Enterobacteria* phage Mu	1WRU_A	90	82.65	23
39	196	Small distal protein	*Escherichia* phage T5	6F2M_D	191	99.96	4.2e-30
40	1266	Large distal protein (RBP?)	*Salmonella* phage vB_SenMS16	6F45_C	125	99.25	7.3e-14

Table S2. Genes and predicted gene products (predicted by HMMER and BLASTp analysis) of the novel phage JK16.

Gene			Gene product					
Orf no.	Base range	Strand (+ forward, - reverse)	Size (no. of aa)	Predicted function	Organism / virus	Accession number	Aligned positions	E-value
1	1..930	-	309	DNA primase	*Escherichia* phage vB_EcoS-IME253	APU93271.1	297	2.6E-87
2	986..1462	-	158	Transcriptional regulator	*Salmonella* phage vB_SenS_PHB07	AVQ09770.1	139	3.7E-65
3	1550..3547	+	665	ATP-dependent helicase	*Escherichia coli*	STH69015.1	498	9.3E-189
4	3547..3966	+	139	VRR-NUC domain-containing protein; nuclease	*Clostridium sp. AM09-51*	WP_118366912.1	114	3E-39
5	4047..4772	+	241	DNA N-6-adenine methyltransferase	*Klebsiella pneumoniae*	SSH28087.1	212	4.5E-67
6	4777..5034	+	85	Hypothetical protein	*Shigella* phage pSf-1	YP_008059729.1	72	3.7E-34
7	5110..5289	+	59	Hypothetical protein	*Shigella* phage pSf-1	YP_008059730.1	59	4E-18
8	5279..5521	+	80	Hypothetical protein	*Escherichia* phage vB_Eco_swan01	SMH63962.1	80	5E-51
9	5466..6731	+	421	Phosphoesterase	*Escherichia* phage ECBP5	YP_009146399.1	372	4.3E-141
10	6877..7092	+	71	Holin	*Escherichia* phage vB_EcoS-IME253	APU93209.1	71	1.4E-33

11	7092..7583	+	163	Lysozyme	*Shigella sonnei*	WP_072107586.1	157	6.9E-84
12	7580..7975	+	131	Putative spanin	*Klebsiella* phage vB_KpnS_KpV522	AOZ65328.1	130	4.5E-48
13	7975..8130	+	51	Hypothetical protein	No match	-	-	-
14	8413..8808	-	131	Hypothetical protein	*Escherichia* phage vB_Eco_SLUR29	VUE36004.1	131	7E-93
15	8805..10385	-	526	DNA primase	*Candidatus Berkiella aquae*	KRG21871.1	526	4.9E-153
16	10454..1675	-	73	Hypothetical protein	*Escherichia* phage vB_Eco_swan01	SMH63954.1	73	3E-44
17	10662..11183	-	173	HNH endonuclease	*Idiomarina zobellii*	SDF27146.1	163	2.4E-36
18	11147..11374	-	75	Hypothetical protein	*Citrobacter* virus Stevie	YP_009148752.1	72	4.9E-33
19	11446..12156	-	236	DNA methylase	*Shewanella* sp. phage 3/49	YP_009103945.1	236	1.0E-77
20	12167..12391	-	74	Hypothetical protein	*Klebsiella* phage KOX1	ARM70371.1	74	1.5E-32
21	12388..12504	-	38	Hypothetical protein	*Escherichia* phage vB_EcoS-95	ASV44824.1	38	8.6e-25
22	12546..12773	-	75	Hypothetical protein	*Escherichia* phage vB_EcoS-95	ASV44825.1	75	2E-47
23	12882..13094	-	70	Hypothetical protein	*Escherichia* virus vB_Eco_mar001J1	VCU43720.1	70	4.3E-38
24	13094..13291	-	65	Hypothetical protein	*Escherichia* phage	QBQ81534.1	65	1.7E-37

					vB_EcoS_G29-2			
25	13367..13630	-	87	Hypothetical protein	*Escherichia* phage vB_Eco_swan01	SMH63947.1	88	4.7E-55
26	13857..14066	-	69	Hypothetical protein	*Escherichia* phage vB_Eco_SLUR29	VUE35992.1	69	5E-43
27	14054..14320	-	88	Hypothetical protein	*Escherichia* phage vB_Eco_swan01	SMH63944.1	88	4E-58
28	14320..14592	-	90	Hypothetical protein	*Escherichia* phage vB_Eco_swan01	SMH63943.1	90	3E-60
29	15269..15451	+	61	Hypothetical protein	*Shigella* phage pSf-1	YP_008059755.1	105	5e-16
30	15854..16318	+	154	Hypothetical protein	*Salmonella* phage YSP2	ATW57845.1	157	3E-36
31	16315..16587	+	90	DUF4884 domain-containing hypothetical protein	*Klebsiella pneumoniae*	WP_119177099.1	91	4E-12
32	16830..17012	+	60	Hypothetical protein	*Escherichia* phage vB_Eco_swan01	SMH63940.1	60	4E-34
33	17028..18152	+	374	Hypothetical protein	*Escherichia* phage vB_Eco_swan01	SMH63939.1	297	4E-149
34	18229..18381	+	50	Hypothetical protein	*Escherichia* phage vB_EcoS_SH2	ARW57197.1	50	4.6E-30
35	18451..18672	+	73	Hypothetical protein	*Escherichia* phage vB_EcoS_G29-2	QBQ81460.1	73	8.6E-49
36	18669..18914	+	81	Hypothetical protein	*Escherichia phage*	ASV44844.1	81	6.7E-56

					vB_EcoS-95			
37	18901..19122	+	73	Hypothetical protein	*Escherichia* phage vB_Eco_swan01	SMH63936.1	73	4.6E-51
38	19134..19673	+	179	Polynucleotide kinase	*Escherichia* phage vB_EcoS-95	ASV44846.1	177	1.5E-57
39	19670..19831	+	53	Hypothetical protein	*Escherichia* phage vB_Eco_swan01	SMH63934.1	53	2E-31
40	19904..20167	+	87	Hypothetical protein	*Escherichia* phage vB_Eco_swan01	SMH63933.1	87	1E-56
41	20164..20348	+	61	Hypothetical transmembrane protein	*Enterobacteria* phage vB_EcoS_IME347	AWD92206.1	63	1.4E-28
42	20561..21151	+	196	ATP-binding protein	*Escherichia* phage vB_Eco_SLUR29	VUE35983.1	196	9E-138
43	21222..21623	+	133	Hypothetical protein	*Escherichia* phage vB_Eco_swan01	SMH63929.1	133	2E-89
44	21723..21971	+	82	Hypothetical protein	*Escherichia* phage vB_EcoS-95	ASV44854.1	82	3E-53
45	21979..22161	+	60	Hypothetical protein	*Escherichia* phage vB_Eco_swan01	SMH63927.1	60	3E-33
46	22173..22463	+	96	Hypothetical protein	*Shigella* phage pSf-1	YP_008059774.1	92	7.3E-47
47	22539..22751	+	70	Hypothetical protein	*Escherichia* phage vB_EcoS-95	ASV44857.1	70	2E-42
48	22748..22981	+	77	Hypothetical protein	*Escherichia* phage vB_Eco_swan01	SMH63924.1	77	1E-49

49	22978..23292	+	104	Hypothetical protein	*Escherichia* virus TLS	SMH63923.1	104	8E-72
50	23363..23491	+	42	Hypothetical protein	*Shigella* phage pSf-1	YP_008059777.1	42	2E-12
51	23491..23721	+	76	Putative fusion protein	*Escherichia* phage vB_EcoS-95	ASV44861.1	76	4E-50
52	23884..24117	+	77	Hypothetical protein	*Shigella* phage pSf-1	SMH63920.1	77	9E-49
53	24074..24259	+	61	Hypothetical protein	*Salmonella* phage 36	YP_009223428.1	60	5E-23
54	24256..24480	+	74	Hypothetical protein	*Escherichia* phage vB_Eco_swan01	SMH63918.1	74	1E-44
55	24559..25086	+	175	Terminase small subunit	*Escherichia* phage vB_EcoS-95	ASV44865.1	175	1E-125
56	25086..26654	+	522	Terminase large subunit	*Belnapia rosea*	SDB74991.1	424	4.7E-161
57	26725..28017	+	430	Portal protein	*Vibrio* phage vB_VchM-138	YP_007006412.1	396	1.9E-117
58	28014..28769	+	251	Phage head morphogenesis protein	*Rosenbergiella nectarea*	WP_092674814.1	258	7.3E-89
59	28780..29895	+	371	Major head subunit precursor	*Citrobacter* virus Stevie			2.1E-81
60	29908..30417	+	169	Putative zonula occludens toxin	*Salmonella* phage 36	YP_009223443.1	157	3E-57
61	30468..31250	+	260	Scaffolding protein SbcC-like protein	*Salmonella* phage 36	YP_009223444.1	250	5E-31
62	31341..32312	+	323	Major capsid protein	*Faecalibacterium* phage FP_Brigit	AUV56656.1	303	4.0E-105
63	32371..32637	+	88	Hypothetical protein	*Escherichia* phage	ASV44873.1	88	2.1E-37

					vB_EcoS-95			
64	32684..33103	+	139	Hypothetical protein	*Klebsiella* phage PKP126	YP_009284937.1	129	1.8E-60
65	33100..33474	+	124	Hypothetical protein	*Escherichia* phage RES-2009a	ACZ74600.1	124	1.2E-51
66	33464..33907	+	147	Putative tail protein	*Escherichia coli*	WP_137576084.1	142	4E-32
67	33897..34298	+	133	Putative tail protein	*Citrobacter* virus Stevie	YP_009148719.1	129	5E-56
68	34301..34963	+	220	Putative major tail protein	*Salmonella* virus SP126	YP_009617994.1	219	4E-121
69	35063..35380	+	105	Tape measure chaperone	*Escherichia* phage vB_EcoS-95	ASV44879.1	105	5E-72
70	35737..38418	+	893	Tail tape measure protein (tail component of prophage)	*Escherichia coli*	CTT34890.1	782	1.1E-294
71	38421..38771	+	116	Tail protein	*Escherichia* phage vB_EcoS-95	ASV44882.1	116	6E-81
72	38835..39596	+	253	Minor tail protein	*Shigella sonnei*	WP_072120829.1	214	9.3E-134
73	39596..40327	+	243	Tail assembly protein	*Shigella sonnei*	CSQ47716.1	182	6.8E-77
74	40308..40904	+	198	Tail assembly protein	*Haemophilus influenzae*	WP_136439541.1	154	2.3E-82
75	40983..44561	+	1192	Tail protein	*Escherichia* phage vB_Eco_swan01	SMH63897.1	1192	0.0
76	44588..45202	-	204	Hypothetical protein	*Escherichia* phage	SMH63896.1	204	2E-148

					vB_Eco_swan01			
77	45483..45752	-	89	Hypothetical protein	*Escherichia* phage Jahat_MG145	QBZ71356.1	89	1.7E-40
78	46254..47309	+	351	Exodeoxyribonuclease VIII	*Snodgrassella alvi*	WP_100139585.1	264	3.5E-85
79	47385..47642	+	85	Hypothetical protein	*Escherichia* phage vB_Eco_swan01	SMH63892.1	85	1E-52
80	47646..48299	+	217	Recombinase	*Escherichia* virus TLS	YP_001285543.1	221	9.0E-72
81	48343..48795	+	150	Single-stranded DNA-binding protein	*Escherichia* phage vB_Eco_SLUR29	VUE36021.1	150	6E-106
82	48828..49502	-	224	Chaperone of endosialidase	*Escherichia* phage Jahat_MG145	QBZ71352.1	231	5Ee-144
83	49515..49793	-	92	Hypothetical protein	*Escherichia* phage vB_Eco_swan01	SMH63888.1	92	2E-57
84	49793..51748	-	651	Putative tail fiber protein	*Escherichia* virus vB_Eco_mar004NP2	VCU43554.1	547	1.3E-175

Table S3. Putative functions of a few of the JK16 tail structural proteins; the functions were estimated via HHpred software.

orf no.	Product size (number of aa)	Putative function(s)	Organism / virus	ID	Aligned positions	Probability (%)
61	260	Tail tube protein;<br>Cell adhesion domain	*Escherichia* phage T5;<br>*Escherichia coli*	5NGJ_B;<br>3NCX_B	100;<br>64	98.02;<br>96.08
68	220	Major tail protein;<br>Portal protein;<br>Baseplate wedge protein	*Enterobacteria* phage lambda;<br>*Bacillus* phage SPP1;<br>*Enterobacteria* phage T4	2K4Q_A;<br>5A21_H;<br>5IV5_o	119;<br>114;<br>103	99.54 ;<br>98.58;<br>67.81
73	243	(Tail associated) lysin	*Streptococcus pyogenes*	5UDN_B	119	96.99
75	1192	Tail protein (RBP?)	Prophage MuSo2	3CDD_E	161	94.62
