# Supplementary figures and images for: A Quest of Great Importance-Developing a Broad Spectrum Escherichia coli Phage Collection
Source: Viruses. 2019 Sep 26;11(10):899. doi: 10.3390/v11100899 (PMC6832132; doi:10.3390/v11100899)

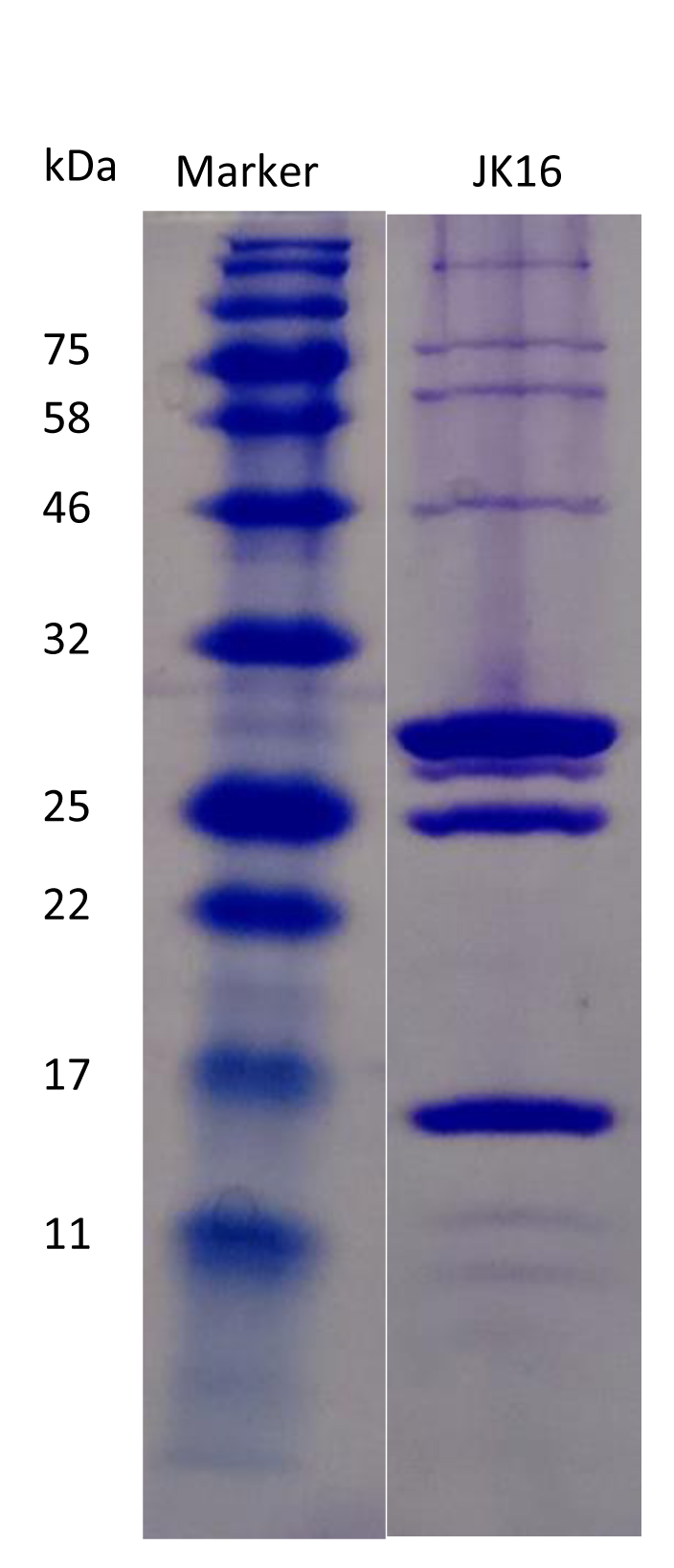

Supplement: Supplementary file 1 [file viruses-11-00899-s001.zip › 7-viruses-533017-suppl/Fig. S1.tiff]
